# Duration and characteristics of persistent headache following aneurysmal subarachnoid hemorrhage

**DOI:** 10.1111/head.14418

**Published:** 2022-11-25

**Authors:** Ben Gaastra, Harry Carmichael, Ian Galea, Diederik Bulters

**Affiliations:** ^1^ Faculty of Medicine University of Southampton Southampton UK; ^2^ Department of Neurosurgery, Wessex Neurological Centre University Hospital Southampton Southampton UK

**Keywords:** headache, migraine, outcome, subarachnoid hemorrhage

## Abstract

**Objective:**

To assess the long‐term frequency, prognosis, and phenotype of persistent headache following aneurysmal subarachnoid hemorrhage (aSAH).

**Background:**

Very little is known about long‐term headache following aSAH with no studies looking beyond 3 years.

**Methods:**

Retrospective analysis comparing aSAH cases to matched controls in the UK Biobank, a prospective cohort study. Headache frequency and phenotype were compared using group comparison tests. The relationship between headache frequency and time was assessed using correlation analysis.

**Results:**

Headache was more frequent following aSAH (aSAH: 258/864 [29.9%] vs. controls: 666/3456 [19.3%], *χ* ^2^ = 45.5, *p* < 0.001) at a median follow‐up of 7.5 years. Headache frequency decreased over time (*R*_S_ = −0.71, *p* = 0.028), affecting 29/58 (50%) patients in the first year and reducing to 13/47 (28%) patients 10 years later. Headache frequency was not related to aSAH severity (*z* = 0.249, *p* = 0.803), treatment (*z* = 0.583, *p* = 0.560), or hydrocephalus (*z* = −1.244, *p* = 0.214). There was a consistently higher frequency of migrainous features following aSAH compared to controls, although this did not reach statistical significance.

**Conclusions:**

Persistent headache is more frequent following aSAH compared to controls in the long term and the prevalence reduces gradually over time. The increased frequency of migrainous features suggests that selected patients with post‐aSAH headache may benefit from migraine treatment.

**Abbreviations**
aSAH: aneurysmal subarachnoid hemorrhage
CSD: cortical spreading depression
ICHD‐3: International Classification of Headache Disorders, 3rd edition
QoL: quality of life
WFNS: World Federation of Neurological Surgeons

## INTRODUCTION

Aneurysmal subarachnoid hemorrhage (aSAH) is a rare but devastating form of stroke which most commonly presents with headache. The International Classification of Headache Disorders, 3rd edition (ICHD‐3)^1^ classifies aSAH‐related headache as either acute (ICHD‐3 6.2.2: Acute headache attributed to non‐traumatic subarachnoid hemorrhage) or chronic (ICHD‐3 6.2.4.2: Persistent headache attributed to past non‐traumatic subarachnoid hemorrhage, which occurs when the headache persists for >3 months).

In the only cohort study of persistent headache after aSAH (*n* = 217), the ictal headache improved continuously over a 12‐month period.^2^ Two further cross‐sectional studies reported that 32.3% (*n* = 405) and 40.9% (*n* = 93) of aSAH cases had headache at 12 and 32.6 months, respectively, with significant negative impact on quality of life (QoL).^3^, ^4^ Beyond these studies, the phenotype, chronicity, and prognosis of persistent headache following aSAH remain poorly defined.

The pathophysiological mechanisms underlying persistent headache following aSAH are not well understood. Potential mechanisms contributing to headache include direct stretch and chemical irritation of the meninges by blood, a neuro‐inflammatory response,^5^ and vascular hyperreactivity including vasospasm.^6^ Cortical spreading depression (CSD) is linked to migraine aura^7^ and has been demonstrated following aSAH.^8^ As CSD has been implicated in both migraine and aSAH, with evidence that it may play a role in initiating headache,^7^ we hypothesized that persistent headache following aSAH may have migrainous features.

The aims of this study were to:

1. Compare the frequency of persistent headache between aSAH and controls at long‐term follow‐up
2. Assess whether and how post‐aSAH headache resolves over time
3. Establish the prevalence of migrainous features in the phenotype of persistent headache following aSAH.

## METHODS

This was a retrospective analysis of cases and matched controls using data from the UK Biobank, a prospective cohort study (application ID 49305). The UK Biobank is a major biomedical database^9^ which recruited 502,497 participants aged 40–69 with informed written consent between 2006 and 2010. Participants attend an initial assessment center visit and were then followed up in person at assessment centers and using multiple online questionnaires. The UK Biobank also obtains data from regular health care record searches dating from before recruitment to the present day. The study is reported according to the STROBE statement and has UK national REC (16/NW/0274) and institutional (University of Southapmton, ERGO 49253) ethical approval.

### aSAH and control individuals

aSAH cases were identified from the UK Biobank using ICD‐9 (data field 41271) and ICD‐10 (data field 41270) codes, self‐reported medical conditions (data field 20002), and primary care data (data field 42040). Consequently, the aSAH could have occurred before or after initial recruitment to the UK Biobank. Individuals were excluded if the subarachnoid hemorrhage was non‐aneurysmal in nature or if they had any traumatic injury within 30 days before/after diagnosis of aSAH (see Table S1 in the Supporting Information for inclusion/exclusion codes). Individuals were included if they had data available on headache outcome (see below).

A single matched control population was generated from the UK Biobank using propensity score matching with a nearest neighbor method and a case:control ratio of 1:4. Control individuals were matched according to variables known to influence headache following aSAH: sex and age at time of follow‐up. Individuals missing headache outcome, age, or sex data were excluded from matching.

### Headache outcome

The question “In the last month have you experienced headache that interfered with usual activity?” (data field 6159, Table S2) was used to assess headache frequency at the first assessment center visit following diagnosis of aSAH. Individuals who answered “yes” were subsequently asked whether they had headache for greater than 3 months (data field 3799).

Migrainous features were assessed in a subset of the above participants who had completed a separate online follow‐up questionnaire focusing on pain (Table S2). Migraine‐type headache was defined as the presence of at least two of the following features: unilateral, throbbing, and moderate‐to‐severe and worse on exertion (data fields 120058‐61), plus at least one feature among nausea, photophobia, and phonophobia (data fields 120062‐64). Individual migrainous features were dichotomized with the feature considered present if reported for either “half the time or more” or “less than half the time,” and absent if the feature was “rarely” or “never” present. Aura was defined as the presence of spreading visual or sensory symptoms preceding or near headache onset (data fields 120065‐68), and prodrome was defined as tiredness, yawning, concentration problems, changes in mood or appetite, irritability, neck stiffness, light or sound sensitivity before or near the onset of headaches (data field 120069). All headache outcomes were considered as binary variables for analysis.

### Statistical analyses

Descriptive statistics of the cohorts were reported using percentages, means (± standard deviation), and medians with interquartile range. The mean was reported when there was no evidence of significant outliers on a histogram; otherwise, the median was reported. Two‐tailed hypothesis testing was performed. The chi‐square test was used to compare binary outcomes and Spearman's rank correlation coefficient used to assess the relationship between headache frequency and time. A *p* value of <0.05 was considered significant with Bonferroni correction for multiple testing applied separately to headache frequency and phenotype. All analyses were performed in R (R Foundation for Statistical Computing).

## RESULTS

**FIGURE 1:**
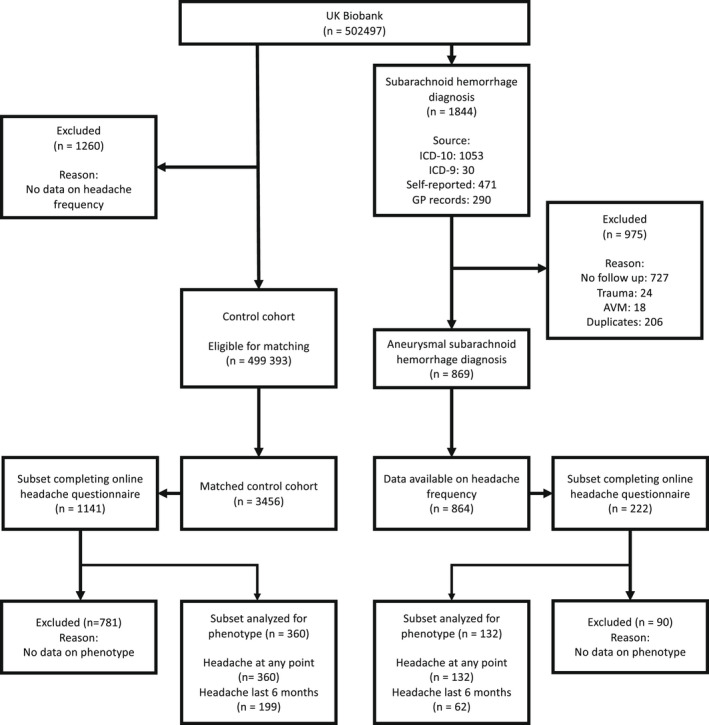
Flow chart for inclusion of aneurysmal subarachnoid hemorrhage and control cohorts from the UK Biobank. Mean standard difference following matching was zero. AVM, arteriovenous malformation.

A total of 869 aSAH cases were identified of which 864 had headache frequency data available, and were matched with 3456 control individuals (see Figure 1 and Table S3 for demographics).

### Headache frequency

More aSAH cases reported headache in the last month that interfered with usual activities compared to the matched control cohort (aSAH: 258/864 [29.9%] vs. controls: 666/3456 [19.3%], *χ* ^2^ = 45.5, *p* < 0.001; Table 1A) at a median follow‐up of 7.5 years. Of these individuals who reported headache, significantly more aSAH cases reported that the headache lasted for more than 3 months (aSAH: 159/252 [63.1%] vs. controls: 302/642 [47.0%], *χ* ^2^ = 18.0, *p* < 0.001; Table 1A). Within the aSAH cases, length of stay (a surrogate marker^10^ of the World Federation of Neurological Surgeons' [WFNS] grade which is not available in the UK Biobank), aneurysm treatment (endovascular/surgical), and hydrocephalus (a known cause of post‐ictal headache) were not significant predictors of headache following aSAH in logistic regression modeling (*z* = 0.249, *p* = 0.803; *z* = 0.583, *p* = 0.560; and *z* = −1.244, *p* = 0.214, respectively).

**TABLE 1:** Comparison of frequency of headache, migraine, aura, and prodrome between aSAH and control cohorts.

		aSAH cohort (*n* = 864)	Control cohort (*n* = 3456)	*χ* ^2^	*p* value
Headache frequency	**A. In the whole study population**				
	Headache in last month that interfered with your usual activities (data field 6159)	258/864 (29.9%)	666/3456 (19.3%)	45.5	<0.001*
	Headache for > 3 months (data field 3799)	159/252 (63.1%)^a^	302/642 (47.0%)^a^	18.0	<0.001*
Headache phenotype	**B. In those reporting a headache at any time**				
	Migraine‐type headache	84/132 (63.6%)	199/359 (55.4%)	2.34	0.127
	**C. In those reporting a headache in the last 6 months**				
	Migraine‐type headache	42/62 (68%)	118/199 (59.3%)	1.09	0.297
	Aura	31/62 (50%)	86/199 (43.2%)	0.63	0.428
	Prodrome	43/62 (69%)	128/198 (64.6%)^b^	0.28	0.597
	Prodrome or aura	48/62 (77%)	146/199 (73.4%)	0.22	0.637
	**D. In those reporting a migraine‐type headache in the last 6 months**				
	Aura	25/42 (59%)	65/118 (55.1%)	0.10	0.751
	Prodrome	34/42 (81%)	86/117 (73.5%)^b^	0.57	0.451
	Prodrome or aura	37/42 (88%)	96/118 (81.4%)	0.58	0.446

*Note*: Migraine type headache was defined as the presence of at least two of the three features of unilateral, throbbing, and moderate‐to‐severe and worse on exertion, plus at least one feature among nausea, photophobia, and phonophobia. Aura was defined as the presence of spreading visual or sensory symptoms preceding or near headache onset, and prodrome was defined as tiredness, yawning, concentration problems, changes in mood or appetite, irritability, neck stiffness, light or sound sensitivity before or near the onset of headaches. Bonferroni correction was applied separately to headache frequency and phenotype domains with the *p* value threshold of significance as <0.025 (0.05/2) and <6.25 × 10^−3^ (0.05/8), respectively.

^a^ There were 6 aSAH cases and 24 control individuals with missing data for determining prodrome status.

^b^ 1 individual was missing data on prodrome.

* *p* < 0.02.

### Headache time course

**FIGURE 2:**
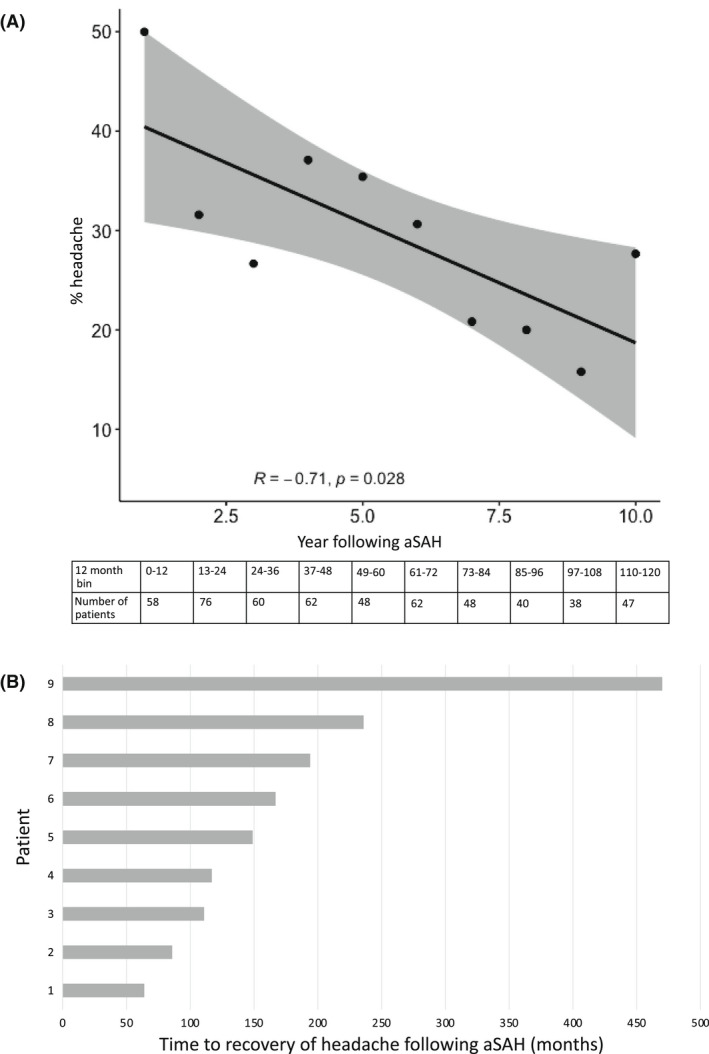
(A) Change in frequency of headache over time, divided into 12 month bins up to 10 years following aneurysmal subarachnoid hemorrhage (aSAH). (B) Time to recovery of persistent post‐aSAH headache, for the 9 out of 21 individuals who were assessed at least twice in the UK Biobank allowing recovery to be recorded.

When patients were binned into annual groups according to time of follow‐up after aSAH the frequency of headache negatively correlated with time, *R*_S_ = −0.71 (*p* = 0.028; Figure 2A and Table S4), decreasing from 29/58 (50%) in the first year to 13/47 (28%) 10 years later. The mean age at follow‐up of aSAH cases increased form 55.9 years to 59.3 years over these 10 annual groups. Twenty‐one individuals with post‐aSAH headache had data on headache frequency at two timepoints. Headache resolved in 9/21 (43%) individuals (Figure 2B); the median time to recovery of headache was 149 months from aSAH.

### Headache phenotype

A total of 132 aSAH cases and 359 controls had data on headache phenotype from the online questionnaire (Figure 1). Migraine‐type headache was more frequent in aSAH cases versus controls, among those with a headache at any time (Table 1B). When limiting the same analysis to those who reported headache in the last 6 months, migraine‐type headache was also more frequent after aSAH (Table 1C). There was also a consistent higher frequency of aura and/or prodrome in aSAH cases compared to controls in those with any type of headache (Table 1C), as well as those with a migraine‐type headache (Table 1D), in the last 6 months.

## DISCUSSION

Headache interfering with usual activity and impairing QoL is more common following aSAH compared to controls, at a median of 7.5 years, and is more likely to be chronic in nature (lasting more than 3 months). The only previous study of headache late after aSAH showed that up to 40.9% of individuals report headache, negatively impacting QoL, at 32.6 months.^4^ Our study builds on this by increasing both the sample size and duration of follow up. Headache becomes less prevalent with time, affecting 50% in the first year post‐aSAH, but reducing to 28% 10 years later. This reduction likely represents resolution of headache over time and decreasing frequency of headache with older age following aSAH.^11^ In the subset of aSAH patients with repeated headache outcome measures, 9/21 (43%) showed complete recovery of headache at a median follow‐up of 149 months. Taken together these results are consistent with previous studies which show improvement in headache over time.^2^ These findings will help with setting patient expectations and emphasize the importance of understanding and managing headache symptoms in the long term as they can take many years to resolve.

Migraine‐type headache was more common following aSAH compared to controls. Aura and/or prodrome were also more common regardless of whether the reported headache was migraine‐type or not. CSD occurs in migraine aura, may play a role in the initiation of headache,^7^ and has been demonstrated in the brain following aSAH.^8^ As aura is a dominant feature in headache following aSAH it suggests CSD may play a role in the pathophysiology of persistent headache following aSAH. This has potential therapeutic implications as standard pharmacological management such as acetaminophen ± opioids for post‐aSAH headache does not significantly improve the headache.^12^ Additional therapies such as magnesium have been shown to reduce headache pain scores although the change was not clinically significant.^13^, ^14^ Migraine preventative treatments, including those which have been shown to influence CSD in migraine, may therefore offer superior analgesia for persistent headache following aSAH.

### Limitations

In the UK Biobank information on a number of factors which may be relevant to headache following aSAH is absent, including WFNS grade, presence of preexisting headache/migraine, and radiological severity of aSAH. In addition, this study does not use validated headache questionnaires which may limit the translation of these results to external datasets. Future studies should include the missing variables and utilize a validated headache questionnaire when analyzing persistent headache after aSAH.

The UK Biobank and this study are biased towards individuals with good outcome as participants are required to attend detailed assessment center visits following aSAH, favoring better performing individuals with fewer neurological sequelae. In addition, in this study only a subset of individuals attended assessment centers twice after aSAH or completed an online questionnaire, which again is likely to select for individuals with better outcome, introducing selection bias into the headache time course and phenotype analysis. The smaller sample size in the phenotype analysis also limits the power of this study to compare phenotype between cases and controls. Selection bias is a feature of the UK Biobank but is not necessarily a major limitation of this study. Anecdotally, patients with the best clinical outcome following aSAH are more likely to be bothered by symptoms such as headache in comparison to poorer outcome individuals who are often preoccupied with more severe neurological deficits. Therefore, the information generated by this study is most likely to be applied to individuals with better outcome, as included in this study. Future prospective studies should include larger sample sizes and multiple serial time points to provide greater insight into persistent headache after aSAH.

## CONCLUSIONS

Persistent headache occurs in the long term following aSAH. Although it may last for years, it gradually improves over time. In some cases, the post‐aSAH headache has migrainous features and while underpowered, this study has provided the data needed for larger prospective studies to confirm these findings and to warrant clinical trials of migraine treatment for selected patients with a post‐aSAH headache with migrainous features.

## AUTHOR CONTRIBUTIONS

*Study concept and design*: Ben Gaastra, Harry Carmichael, Ian Galea, Diederik Bulters. *Analysis and interpretation of data*: Ben Gaastra, Harry Carmichael, Ian Galea, Diederik Bulters. *Drafting of the manuscript*: Ben Gaastra, Harry Carmichael. *Revising it for intellectual content*: Ben Gaastra, Harry Carmichael, Ian Galea, Diederik Bulters. *Final approval of the completed manuscript*: Ben Gaastra, Harry Carmichael, Ian Galea, Diederik Bulters.

## FUNDING INFORMATION

BG is funded by the Royal College of Surgeons, Society of British Neurological Surgeons, Barrow Foundation, and Guarantors of Brain in addition to the Institute for Life Sciences, University of Southampton.

## CONFLICT OF INTEREST

The authors declare that there are no conflicts of interest.

## Supporting information


Appendix S1

## Data Availability

The data that support the findings of this study are available from the UK Biobank (https://www.ukbiobank.ac.uk) by application.
