# Supplementary material for: Duration and characteristics of persistent headache following aneurysmal subarachnoid hemorrhage
Source: Headache. 2022 Nov 25;62(10):1376–82. doi: 10.1111/head.14418 (PMC10099612; doi:10.1111/head.14418)
Supplement: Supplementary file 1 — Appendix S1 [file HEAD-62-1376-s001.docx]

SUPPLEMENTARY MATERIAL

| Data field | Code and definition |
| --- | --- |
| Inclusion | |
| Data field 41270  ICD 10 codes | I600         I60.0 Subarachnoid hemorrhage from carotid siphon and bifurcation  I601         I60.1 Subarachnoid hemorrhage from middle cerebral artery    I602         I60.2 Subarachnoid hemorrhage from anterior communicating artery    I603         I60.3 Subarachnoid hemorrhage from posterior communicating artery    I604         I60.4 Subarachnoid hemorrhage from basilar artery    I605         I60.5 Subarachnoid hemorrhage from vertebral artery    I606         I60.6 Subarachnoid hemorrhage from other intracranial arteries    I607         I60.7 Subarachnoid hemorrhage from intracranial artery, unspecified    I609         I60.9 Subarachnoid hemorrhage, unspecified  Ruptured aneurysm |
| Data field 41271  ICD9 | 430 Subarachnoid hemorrhage  4309        Subarachnoid hemorrhage |
| Data field 42040  Primary care data | G60 Equates to ICD-10 code I609  G600 Equates to ICD-10 code I607  G601 Equates to ICD-10 code I600  G602 Equates to ICD-10 code I601  G603 Equates to ICD-10 code I602  G605 Equates to ICD-10 code I604  G606 Equates to ICD-10 code I605  G60X Equates to ICD-10 code I607  G60z Equates to ICD-10 code I609  Gyu60 Equates to ICD-10 code I606  Gyu6E Equates to ICD-10 code I607  X00Df Equates to ICD-9 code 430  X00Dg Equates to ICD-10 code I609  X204F Equates to ICD-10 code I609  Xa01c Equates to ICD-10 code I606  Xa01h Equates to ICD-10 code I601  Xa01i Equates to ICD-10 code I606  Xa01j Equates to ICD-10 code I602  Xa01k Equates to ICD-10 code I603  Xa01l Equates to ICD-10 code I604  Xa01m Equates to ICD-10 code I606  Xa01o Equates to ICD-9 code 430 |
| Data field 20002  Self-reported medical conditions | 1086        Subarachnoid hemorrhage |
| Exclusion | |
| Data field 41270  ICD 10 codes | Q282       Q28.2 Arteriovenous malformation of cerebral vessels    Q283       Q28.3 Other malformations of cerebral vessels    S-T            Injury, poisoning and certain other consequences of external causes    V,W,X      External causes of morbidity and mortality |
| Data field 41271  ICD9 | 74780      Arteriovenous aneurysm of brain    74781      Other anomalies of cerebral vessels    800-900   Trauma and injury |
| Data field 42040  Primary care data | P7y01 Equates to ICD-10 code Q282  P7y02 Equates to ICD-10 code Q283  S, U Equates to ICD-10 codes S,T,V,W,X and ICD-9 codes 800-900 |

**Supplementary table 1.** Inclusion and exclusion codes for aSAH cases in the UK Biobank. ICD: International Classification of Diseases.

|  | Data field | Description |
| --- | --- | --- |
| Headache frequency | 6159  3799 | Headache in last month that interfered with your usual activities  Headache for greater than 3 months |
| Headache phenotype | 120045  120058  120059  120060  120061  120062  120063  120064  120065  120066  120067  120068  120069 | Headache in past six months  Pain worst on just one side when headaches were at their worst  Pain pounding, pulsating or throbbing when headaches were at their worst  Pain moderate or severe when headaches were at their worst  Pain made worse by routine activities when headaches were at their worst  Felt sick or being sick when headaches were at their worst  Bothered by light when headaches were at their worst  Bothered by sound when headaches were at their worst  Visual changes before or near the onset of headaches  Visual changes develop slowly before or near the onset of headaches  Tingling or numbness in face, arms or legs before or near the onset of headaches  Tingling and/or numbness spreads slowly before or near the onset of headaches  Develop tiredness, yawning, concentration problems, changes in mood or appetite, irritability, neck stiffness, light or sound sensitivity before or near the onset of headaches |

**Supplementary Table 2.** Headache data fields from the UK Biobank used in this study.

|  | aSAH cohort | Control cohort |
| --- | --- | --- |
| Total sample size, n  Subset completing online questionnaire | 864  222 (25.7%) | 3456  1141 (33.0%) |
| Age at time of follow up  Mean (±SD) years | 58 (± 7.2) | 58 (± 7.1) |
| Sex  Male  Female | 352 (40.7%)  512 (59.3%) | 1408 (40.7%)  2048 (59.3%) |
| Time to first follow up  Median (IQR) months | 91 (124) | - |
| Length of stay  Median (IQR) days  Missing | 7 (12)  325 (37.6%) | -  - |
| Hydrocephalus | 44 (5.1%) | - |
| Aneurysm treatment  Surgical  Endovascular  Missing | 159 (18.4%)  128 (14.8%)  557 (66.8%) | -  -  - |

**Supplementary Table 3.** Demographics of aSAH cases and the matched control cohort. The presence of hydrocephalus was defined using Office of Population Censuses and Surveys Classification of Interventions and Procedures, version 4 (OPCS-4) codes A201 (drainage of ventricle of brain) and A124 (creation of a ventriculoperitoneal shunt) from data field 41272 at time of or within the year following diagnosis of aSAH. Treatment was also defined using OPCS-4 codes with L332 (clipping of aneurysm of cerebral artery) coded as surgical, and O01 (transluminal coil embolization of aneurysm of artery) and L351 (percutaneous transluminal embolization of cerebral artery) coded as endovascular. SD: standard deviation. IQR: interquartile range.

| Time to follow up following aSAH (month) | Total sample size | Number reporting headache |
| --- | --- | --- |
| 0-12 | 58 | 29 (50%) |
| 13-24 | 76 | 24 (32%) |
| 25-36 | 60 | 16 (27%) |
| 37-48 | 62 | 23 (37%) |
| 49-60 | 48 | 17 (35%) |
| 61-72 | 62 | 19 (31%) |
| 73-84 | 48 | 10 (21%) |
| 85-96 | 40 | 8 (20%) |
| 97-108 | 38 | 6 (16%) |
| 110-120 | 47 | 13 (28%) |

**Supplementary Table 4.** Number of individuals reporting headache (assessed by data filed 6159) divided into 12 month bins up to 10 years.
